# Community Survey after Rabies Outbreaks, Flagstaff, Arizona, USA

**DOI:** 10.3201/eid1806.111172

**Published:** 2012-06

**Authors:** Andrea M. McCollum, Jesse D. Blanton, Robert C. Holman, Laura S. Callinan, Steven Baty, Randy Phillips, Michael Callahan, Craig Levy, Ken Komatsu, Rebecca Sunenshine, David L. Bergman, Charles E. Rupprecht

**Affiliations:** Centers for Disease Control and Prevention, Atlanta, Georgia, USA (A.M. McCollum, J.D. Blanton, R.C. Holman, L.S. Callinan, S. Baty, C.E. Rupprecht, R. Sunenshine);; Arizona Department of Health Services, Phoenix, Arizona, USA (S. Baty, C. Levy, K. Komatsu);; Coconino County Public Health Services District, Flagstaff, Arizona, USA (R. Phillips, M. Callahan);; US Department of Agriculture, Phoenix (D.L. Bergman)

**Keywords:** rabies virus, lyssavirus, rabies, health knowledge, attitudes, practice, outbreak, epizootic, community survey, viruses, zoonosis, Arizona, United States, USA, translocation, wild animals, wildlife, education

## Abstract

Flagstaff, Arizona, USA, experienced notable outbreaks of rabies caused by a bat rabies virus variant in carnivore species in 2001, 2004, 2005, 2008, and 2009. The most recent epizootic involved transmission among skunk and fox populations and human exposures. Multiple, wide-ranging control efforts and health communications outreach were instituted in 2009, including a household survey given to community members. Although the Flagstaff community is knowledgeable about rabies and the ongoing outbreaks in general, gaps in knowledge about routes of exposure and potential hosts remain. Future educational efforts should include messages on the dangers of animal translocation and a focus on veterinarians and physicians as valuable sources for outreach. These results will be useful to communities experiencing rabies outbreaks as well as those at current risk.

## Abstract 2

Educational outreach should inform the public about dangers of translocation of wild animals and general aspects of rabies.

More than 90% of rabies cases in the United States are in wild animals. Most reported cases of rabies occur among carnivores, including raccoons, skunks, and foxes, in addition to many bat species. Despite the elimination of canine rabies virus variants in the United States, domestic animals, including cats and dogs, are infected each year from exposures to rabid wildlife. In addition, ≈2–4 human rabies cases are reported each year in the United States (*1*), and exposure to rabid animals or animals suspected of being rabid is common, with ≈35,000–38,000 persons receiving rabies postexposure prophylaxis (PEP) each year (*1**,**2*). One of the primary methods for rabies prevention and control is practical and accurate public health information. Recognition of the signs and severity of rabies, exposure routes, behavioral and environmental risk factors, and appropriate domestic animal welfare are critical messages for disease prevention and require appropriate public education for persons of all ages (*3**,**4*).

Rabies virus is generally transmitted among members of the same species, and specific rabies virus variants are associated geographically with independent reservoir species. Spillover of rabies virus variants from 1 species to another occurs, but sustained transmission of such variants in nonreservoir species is rare (*4*). The area around Flagstaff, Arizona (Coconino County), USA, was free of sustained rabies virus transmission until 2001, when a spillover of a bat rabies virus variant was followed by a suspected host shift, with increased transmission in striped skunk (*Mephitis mephitis*) populations (*5*). Control measures were launched to halt rabies spread in skunks and limit the potential for human exposures (*6*). These efforts appeared to control rabies spread in skunk populations until 2004, when 5 striped skunks and 1 gray fox (*Urocyon cinereorgenteus*) were diagnosed as rabid, and rabies was confirmed in an additional striped skunk, a gray fox, and a feral cat (*Felis catus*) in 2005 (*5*). Rabies was quiescent after this resurgence in 2004/2005 until fall 2008 when the disease was confirmed in several gray foxes and striped skunks (*4*). The establishment of rabies in fox populations was troubling because the extensive home range of foxes threatened its containment in the Flagstaff area. Given the size of this epizootic, the potential for spread to other areas, and several notable human exposures, a large, interagency effort was launched to control the resurgence of rabies in Flagstaff.

In October 2009, a survey was distributed to Flagstaff households in an area where rabid animals had been captured in 2008 and 2009. This area also had a history of rabies epizootics since 2001 (*5**,**6*). Attitudes and practices regarding management of exposure to domestic and wild animals are essential to define in areas where persons and their pets may have an increased chance of coming into contact with a rabid animal. An assessment of community knowledge of rabies and interactions with animal reservoirs can help target educational messages during seasonal disease peaks or at the beginning of an epizootic. We present an update on the most recent outbreak and the results of a community survey in Flagstaff.

## Methods

### Data Sources and Survey Design

Surveillance data for Coconino County of the numbers of rabid animals identified during 2000–2009, were obtained from the Arizona Department of Health Services. Emergency department admission data, in which the chief complaint included animal bites during 2005–2009, were obtained from the infection control office for the Flagstaff Medical Center.

Addresses of all households in the quarantine area of Flagstaff were provided by the Coconino County Public Health Services District. Occupancy status of the households was not available, and names associated with each address were permanently removed and not shared for the mailings. In October 2009, surveys were mailed to all households, and 1 adult from each household was asked to complete the survey. Respondents could complete the survey online or by an included paper-based form and returned in a prepaid envelope by mail. The surveys were anonymous and were not linked to a name or address. Educational material on rabies was not included, but for more information, respondents could request printed materials on a separate request form and were directed to the rabies website of the Centers for Disease Control and Prevention (CDC) (Atlanta, GA, USA) (*7*). The community survey was determined to be public health nonresearch by CDC. The survey elicited information from respondents on knowledge of rabies, the Flagstaff rabies outbreak, practices regarding domestic and wild animals, and adherance to quarantine restrictions. Survey questions are included in Table A1.

### Data Analysis

A general definition of “knowledge of rabies” was defined as the answer of “yes” when the respondent correctly identified that bites, scratches, and saliva were modes of rabies virus transmission and also identified 1 other incorrect mode of transmissions as a mode of transmission or did not identify any incorrect mode of transmission. A more specific definition of “knowledge of rabies in Flagstaff” was defined as “yes” if the respondent knew about the outbreak in Flagstaff and knew the 3 main animals which had rabies in Flagstaff (bat, skunk, and fox).

Univariate and multivariate analyses were conducted for 4 separate resident subpopulations to further characterize the groups. The outcomes of interest were 1) knowledge of rabies in Flagstaff, 2) pet owners, 3) dog owners, and 4) translocators (persons who trapped and moved wild animals on their property). Odds ratios with 95% CIs for individual characteristics were calculated by using logistic regression. Characteristics that were considered associated (p<0.1) with each outcome in the univariate analysis were further assessed through multivariate logistic regression models (*8*).

## Results

### Update on Rabies Epizootic

**Figure 1:**
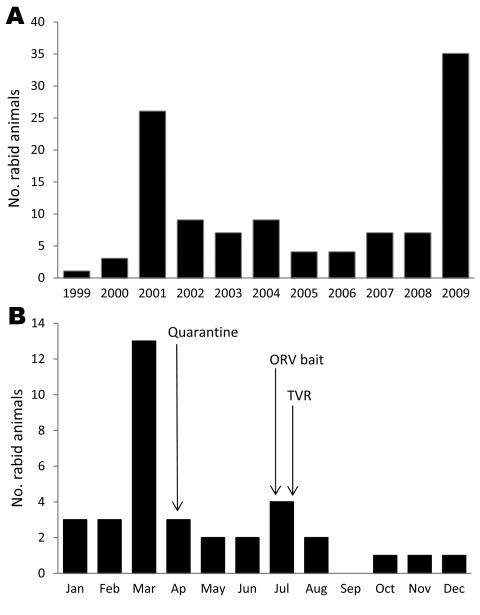
Reported rabid animals, Coconino County, Arizona, USA. A) Number of rabid animals confirmed by laboratory testing, 1999–2009. B) Number of rabid animals during 2009 and response activities. ORV bait, oral rabies vaccination bait; TVR, trap, vaccinate, release campaign.

After a period of quiescence from 2005, another rabies epizootic occurred in Flagstaff during 2008 (Figure 1, panel A). Seven rabid animals were reported in Coconino County, including 2 foxes and 2 skunks (Table 1). No human exposures to rabid animals were reported in 2008, but several companion animal exposures occurred, including 6 cats exposed to rabid bats, 2 dogs exposed to rabid skunks, and 1 dog exposed to a rabid fox. In 2009, Coconino County reported 35 rabid animals. All rabies viruses typed from the immediate Flagstaff area (from 14 foxes and 1 ringtail cat) were identified as a bat rabies virus variant. Two human exposures to rabid animals were identified in 2009: one person had been bitten by a rabid fox and the other person had been bitten by a rabid skunk. Also, 3 companion dog exposures to rabid foxes were reported. A review of Emergency Department discharge data at Flagstaff Medical Center specified a total of 88 animal bite–related admissions during 2005–2009. In 2009, 25 animal bite–related admissions were recorded, and 12 (48%) persons received PEP.

**Table 1:** Annual number of rabid animals confirmed, Coconino County, Arizona, USA, 2008–2010

Species	2008	2009	2010
Bat	1	4	4
Bobcat	1	0	0
Coyote	0	1	0
Fox	3 (2*)	24 (14*)	0
Ringtail	0	1 (1*)	0
Skunk	2 (2*)	5	0
Total	7 (4*)	35 (15*)	4

*Number of animals positive for brown bat rabies virus variant.

**Figure 2:**
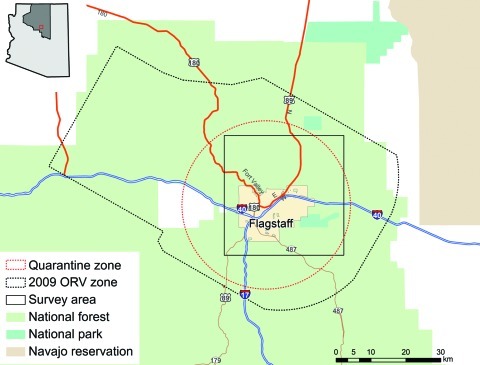
Flagstaff, Arizona, USA, survey area in relation to quarantine and oral rabies vaccination (ORV) zones.

The number of rabid animals and control measures that were initiated in Coconino County in 2009 are shown in Figure 1, panel B. During July 21–24, the US Department of Agriculture, Animal and Plant Health Inspection Service, Wildlife Services, distributed 140,000 oral rabies vaccination (ORV) baits containing a vaccinia–rabies glycoprotein vaccine (Merial, Duluth, GA, USA) by air and ground over a 191-km^2^ area in Coconino County, targeting gray foxes (Figure 2). The vaccinia–rabies glycoprotein vaccine is not effective for vaccinating skunks against rabies (*9*). A 6-week trap, vaccinate, release campaign targeting skunks was initiated in the eastern portion of Flagstaff at the end of July. Additional control initiatives included prohibiting relocation of nuisance wildlife, comprehensive public education on rabies, rabies vaccine clinics for pets, a leash policy for pets on trails, and quarantine. The quarantine was established from April 7 through September 13, 2009, for a 15-mile-radius area centered on Flagstaff and later expanded to the entire ORV zone. The following measures were mandatory: do not disrupt ORV baits, do not feed wild animals, enclose compost bins and piles, do not leave pet food outside after sundown, confine cats and dogs to an enclosure on the owner’s property, keep pets on a leash when off of the owner’s property, and maintain current rabies vaccination for cats and dogs.

### Demographic and Rabies-related Characteristics of Household Respondents

A total of 3,141 surveys were mailed, but 172 were returned because of an incorrect address or no occupancy of household, and 1,058 were completed and returned (35.6%); 1,039 written and 19 website-based. Most respondents had lived in Flagstaff for ≥10 years (74%), lived in Flagstaff year round (98%), were ≥51 years of age (68%), female (59%), and had at least a college degree (75%). A summary of responses is shown in Table A1.

Most respondents recognized that rabies virus can be transmitted to humans from infected animals through a bite (97%), scratch (73%), or contact with saliva (74%). More than half of respondents thought rabies virus can be transmitted by contact with blood, almost a quarter by contact with an infected animal’s urine or feces, and 13% identified skunk spray as infectious. Most residents were aware that skunks and foxes in Flagstaff may have rabies (89% and 73%, respectively), but only 52% were aware that bats in Flagstaff had rabies. Information about the current outbreak was ascertained by many methods, with newspapers or magazines being the most frequently cited source (78%).

Most (70%) respondents reported that if they were bitten or scratched by a domestic animal they would wash the wound with soap and water and likely seek medical care. More persons indicated they would seek medical care if they had an encounter with a wild (90%) animal than with a domestic (72%) animal. Most respondents indicated they would call one of 3 public agencies (city animal control, county health department, fish and game department) if they saw a sick animal than if they were bitten or scratched by an animal. Sixty residents (6%) reported seeing a sick wild animal on their property in the last 6 months. Of those that specified the type of animal, 21 persons (38%) reported a sick skunk, 20 (36%) a sick fox, and 1 (2%) a sick bat; 38% of responses were listed as “other.” Thirty-six percent of the residents reported doing nothing after seeing the ill animal, while 36% called animal control, and 17% called the county health department.

Seventy-three respondents (7%) reported that they would trap and translocate a nuisance animal themselves. Ninety-five persons (9%) have personally relocated a wild nuisance animal that was on their property. Furthermore, 57% translocated the animals >5 miles from their property. Skunks were the most frequent animal to be translocated (56%).

Eighty-four percent of respondents were aware of the rabies quarantine, and 82% of those stated that they complied with quarantine restrictions. Twelve percent of respondents did not believe the requirement to keep pets on a leash at all times would help prevent rabies exposures.

One half of all households owned dogs, and 29% of all households owned cats (Table 2). The overall proportion of dogs vaccinated was 96%. A higher proportion of outdoor cats (90%) was vaccinated than were indoor (76%) or indoor/outdoor (84%), and the overall reported proportion of cats vaccinated was 81%.

**Table 2:** Characteristics of households that owned pets, Flagstaff, Arizona, USA, 2009

Characteristics	No. (%)
Pet owner	684 (65)*
Dog owner	528 (50)*
Dog(s) quarantined for possible rabies exposure	21 (4)
No. dogs currently	
Mainly indoor	638 (85)
Mainly outdoor	115 (15)
Total	753 (100)
No. dogs with current rabies vaccination	
Mainly indoor	615 (96)†
Mainly outdoor	106 (92)†
Total	721 (96)†
Cat owner	308 (29)*
No. cats currently	
Indoor	261 (51)
Outdoor	31 (6)
Indoor/outdoor	218 (43)
Total	510 (100)
No. cats with current rabies vaccination	
Indoor	199 (76)†
Outdoor	28 (90)†
Indoor/outdoor	184 (84)†
Total	411 (81)†

*Frequency among all survey respondents (n = 1,058). †Proportion vaccinated.

### Rabies Knowledge in Flagstaff, Pet Ownership, Dog Ownership, and Translocation

Persons who had knowledge of rabies in Flagstaff were more likely to have knowledge of other aspects regarding rabies (Table A2), including the quarantine and concern about rabies in Flagstaff; these characteristics were independently associated with knowledge of rabies in Flagstaff (Table 3). Male respondents were more likely to have knowledge of rabies in Flagstaff than female respondents. Multivariate analysis did not identify an independent association between those who have knowledge of rabies in Flagstaff and contact with a nuisance wild animal.

**Table 3:** Multivariate analysis of respondents’ rabies knowledge in Flagstaff, pet ownership, dog ownership, and translocation, with demographic and rabies-related characteristics*

Characteristic	Odds ratio (95% CI)			
	Knowledge of rabies in Flagstaff†	Pet owners	Dog owners	Translocators
Concern about rabies in Flagstaff				
Concerned	2.49 (1.21–5.15)			
Not concerned				
Potential for contact with nuisance wild animal on property				
Yes				16.33 (9.98–26.74)
No				Referent
Aware of quarantine in Flagstaff during 2009				
Yes	4.20 (2.67–6.62)		2.24 (1.55–3.23)	
No	Referent		Referent	
Leash policy prevents pet exposure to rabid animals				
Yes		0.39 (0.23–0.68)	0.27 (0.17–0.44)	
No		Referent	Referent	
Years lived in Flagstaff				
>10				3.73 (1.77–7.86)
<10				Referent
Sex				
F	0.74 (0.56–0.96)			
M	Referent			
Characteristics interaction‡				
Age >60 y				
Potential for contact with sick domestic animal		0.96 (0.56–1.66)	1.04 (0.60–1.83)	
No potential for contact with sick domestic animal		Referent	Referent	
Age <60 y				
Potential for contact with sick domestic animal		2.78 (1.67–4.63)	2.20 (1.46–3.27)	
No potential for contact with sick domestic animal		Referent	Referent	
Women				
Aware of quarantine		5.42 (3.29–8.95)		
Not aware of quarantine		Referent		
Men				
Awa Aware of quarantine		1.37 (0.79–2.39)		
No Not aware of quarantine		Referent		

*Characteristics and interactions significant in the multivariate regression analysis, p<0.05. †Knowledge of rabies in Flagstaff is defined as “yes” if the respondent 1) knew about the outbreak in Flagstaff and 2) knew the 3 main animals that have had rabies in Flagstaff (bat, skunk, fox). ‡Odds ratio estimates for individual terms involved in interaction are not displayed.

Pet and dog owners were more likely to have had contact with a sick domestic animal and to be aware of the rabies quarantine than those who did not own pets (Table A2). Pet ownership in general was associated with knowledge of rabies. In multivariate analyses, pet owners were less likely to believe that the leash policy prevents exposures to rabid animals than non–pet owners. Dog owners were more likely to be aware of the 2009 quarantine (Table 3). There was significant interaction between age group and potential for contact with sick domestic animals, and for respondents <60 years of age, those that indicated potential for contact with a sick domestic animal were more likely to be general pet owners or dog owners. Among women, general pet owners were more likely to be aware of the quarantine than non–pet owners; this association was not seen for men. Men and women were similarly aware of the quarantine (83% and 84%, respectively).

Persons who had translocated nuisance animals were more likely to be male and to not own a pet (Table A2), although these associations did not remain independently significant in the multivariate analysis (Table 3). In the multivariate analyses, those who moved animals from their property were more likely to have a potential for contact with a wild animal and to have lived in Flagstaff for at least 10 years.

## Discussion

An extensive outbreak control and education campaign took place in 2009. As observed in past interventions, the epizootic waned and in 2010 only 4 rabid bats were reported from Coconino County. This decline in rabid animals is likely attributable, in part, to the broad interagency control campaigns. Whether another epizootic will occur in Flagstaff remains to be determined. However, the multiple outbreaks over the last decade have resulted in a substantial change in rabies epizootiology in northern Arizona. The repercussions of a potential perpetuation of a bat rabies virus variant in gray fox populations are a concern, given the wide-ranging movements of these carnivores. In addition, these outbreaks have been associated with an increased number of visits to the emergency department of a local hospital, where 48% of persons with animal bite-related visits required rabies PEP in 2009. Heightened vigilance and continued laboratory-based surveillance are warranted in the immediate vicinity and surrounding areas.

Educational efforts were initiated by Coconino County during the current and previous epizootics (*6*). Residents of Flagstaff have received educational messages about rabies and the existing outbreaks through many methods, which likely had a positive effect on the extent of knowledge retained by community members.

Although residents had a general knowledge of rabies as a disease, a large number of persons did not give correct answers to some general knowledge questions, including routes of exposure and animals that can be infected. These misconceptions have been noted in other surveys (*10**,**11*). Future efforts should consider including information about which animals have been reported as rabid in the community and what animals are susceptible. Furthermore, education efforts should focus on specific exposure routes of concern and address possible misconceptions regarding the infectious nature of other bodily fluids such as blood, urine, feces, or skunk spray. This information could play a key role in reducing public concern about rabies virus exposure from noninfectious routes.

Most respondents reported appropriate medical responses to being bitten or scratched by an animal, which include washing of the wound and seeking medical care. Decisions on the risk for rabies and administration of rabies PEP should be made by medical professionals with consultation from local or state public health professionals (*12*). Information about appropriate actions after animal exposure should be maintained in future outreach materials.

The City of Flagstaff Animal Control and Coconino County Public Health Services District Animal Management Office respond to calls related to wild and domestic animals, while the Arizona Game and Fish Department responds only to calls related to wild animals. In contrast to a large number of respondents (>40%) indicating that they would notify one of the agencies of an ill animal on their property, 32% of persons who had seen an ill animal (including bat, skunk, and fox) did nothing. Regardless of these differences, clear, concise instruction about which agency should be notified would be useful for residents and may help streamline notification.

During the recent rabies outbreak in Flagstaff, human and pet exposures occurred from encounters with rabid foxes and skunks. Rabid animals exhibit aggressive or altered behavior which puts others at risk. However, in some circumstances, human-animal contact is a result of the person initiating contact with the animal. Some respondents indicated that they would put themselves in direct contact with ill or nuisance wild animals, and some have trapped and translocated nuisance animals, primarily skunks. The county provides traps for residents to use, with the request that residents bring trapped animals to animal control. This service increases the likelihood that some residents will 1) come into contact with an unknown animal and 2) may translocate that animal. This analysis identified living in Flagstaff for at least 10 years as a characteristic associated with translocators. Long-term residents may be more aware of traps provided by the county.

Approximately half of respondents who have translocated animals moved the animal to an area >5 miles from their property. Thus, long range movement of reservoirs, possibly outside of the trap-vaccinate-release area, has probably occurred. Consequently, not only does translocation expand the range of an outbreak, but removal of target species could diminish local herd immunity by removal of vaccinated animals. Translocation of animals threatens the success of control programs and the spread of rabies has been attributed to translocation (*13**,**14*). Continued outreach, to the community and nuisance operators, should emphasize the risks of translocation to humans, animal populations, and rabies control programs. No local ordinances address the topic or prohibit translocation in Coconino County. State and local ordinances and enforcement should be considered to prevent translocation of rabies reservoirs.

This study found that pet owners had a basic knowledge of rabies and the quarantine. A recent survey conducted in Texas found that dog owners knew more specific facts about rabies than persons who did not own dogs (*15*). Several respondents in Flagstaff noted learning about the outbreak from their veterinarian. This survey did not assess specifics of veterinarian instruction to pet owners; however, this would be a useful avenue of study (*15*). Dog owners were less likely to believe that a mandatory leash policy would help prevent exposure to rabid animals. Local trails are popular destinations for dog owners, and dogs are frequently taken off the leash on these trails. Outreach about exposures and risks to humans and their pets may be warranted for dog owners in particular.

The households in the quarantine area that participated in this study have a larger number of dogs (0.71 for every household) than the estimated national average of 0.63, and an average number of cats the same as the national average (0.48 per household) (*16*). Whether the high proportion of vaccinated animals found in this survey is a reflection of the demographics of the households or a result of the ongoing outbreak and quarantine regulations, is unknown. Vaccination of dogs, but not cats, is required in Arizona (*1*), and Arizona utilizes the vaccination scheme recommended in the 2008 Compendium of Animal Rabies Prevention and Control which recommends that dogs and cats be vaccinated at 3 months of age, 12 months of age, and a receive a booster every year or every 3 years, depending on vaccine label specifications (*17*). Cats are the leading domestic animal reported with rabies in the United States, and consequently, cats are responsible for a substantial proportion of rabies exposures to persons (*18*). Vaccination of companion animals that have regular human contact is a basic, simple, and critical barrier to exposure. Veterinarians and public health, and animal control personnel should emphasize vaccination of domestic dogs and cats. Continued education and vaccination measures will help alleviate risk to companion animals, and subsequently, to humans.

This study has several limitations. First, only household members in Flagstaff who responded to the survey are characterized in this study, and without characterization of nonresponders, a nonresponse bias cannot be evaluated. Also, not all questions were answered by all respondents. Compared with the 2000 US Census data for Flagstaff (*19*), the survey respondents were older (68% vs. 5.3% >65 years of age), more likely to have a college degree (75% vs. 39.4%), and more likely to be female (59% vs. 50.4%). Taken together, these demographics may have biased the study in regards to rabies knowledge, but these differences are not necessarily correlated with increased rabies knowledge. In addition, this survey was paper-based with the option to respond to an online version. Less than 2% of completed surveys were Internet-based. The results may be biased and reflect a population that is more likely to complete a paper-based survey versus using social media or a survey administered through email. Also, data from factors such as language barriers and social economic status were not collected, and the results may be affected by such factors.

The findings of this study provide helpful information for county public health in support of their community outreach efforts and where additional efforts might be focused. In particular, a focus on reinforcing rabies virus transmission routes and exposure guidelines should help reduce public concern about nonexposure events and possibly reduce inquiries to health authorities about such events. This information will be helpful in the event of a future outbreak in Flagstaff or for reference in surrounding areas, especially if rabies expands outside Flagstaff and Coconino County. Rabies has not been reported on the adjacent Navajo Nation for many years. Additional measures would be necessary to tailor prevention and control activities if rabies was to reemerge in this area. In addition to existing messages distributed by media, local public agencies may wish to bolster their existing internet information for the community, as well as outreach through local veterinarians. Outreach to physicians should also be conducted, to reinforce current Advisory Committee on Immunization Practices recommendations on human rabies prevention and PEP administration, as well as to encourage consultation with local and state public health officials to assist with exposure assessment.

## Appendix

**Table A1:** Univariate analysis of respondents’ rabies knowledge in Flagstaff, pet ownership, dog ownership, and translocation, with demographic and rabies-related characteristics *

Characteristic	No. (%)
Years lived in Flagstaff	
<2	53 (5)
3–9	218 (21)
>10	765 (74)
Live in Flagstaff year-round	
No	25 (2)
Yes	1019 (98)
Sex	
M	424 (41)
F	600 (59)
Age, y	
18–30	43 (4)
31–40	115 (11)
41–50	180 (17)
51–60	317 (31)
61–70	250 (24)
>71	129 (13)
Education	
<High school	9 (1)
High school	54 (5)
Some college	193 (19)
College degree	337 (33)
Graduate or professional degree	422 (42)
Knows what rabies is	
No	42 (4)
Yes	997 (96)
Concerned about rabies in Flagstaff	
No	54 (5)
Slightly	646 (64)
Yes	304 (30)
How severe is rabies?	
Mild	35 (4)
Severe	818 (91)
Do not know	44 (5)
Humans get rabies from an infected animal by†	
Bite	1,030 (97)
Scratch	772 (73)
Touching	173 (16)
Contact with blood	616 (58)
Contact with saliva	781 (74)
Contact with urine or feces	258 (24)
Contact with spray of infected skunk	141 (13)
Do not know	22 (2)
Animal could be infected with rabies†	
Dog	1,040 (98)
Cat	965 (91)
Horse	407 (38)
Livestock	418 (40)
Bat	1023 (97)
Rabbit	691 (65)
Rodent	855 (81)
Wild carnivore	999 (94)
Bird	144 (14)
None of the above	3 (0.2)
Do not know	6 (0.5)
Wild animals that have rabies in Flagstaff†	
Bat	546 (52)
Skunk	943 (89)
Fox	772 (73)
Raccoon	305 (29)
Ringtail	113 (11)
Coyote	266 (26)
Mountain lion	164 (16)
No wild animals have rabies	11 (1)
Response to seeing a sick domestic animal†	
Nothing	26 (3)
Take the animal to a veterinarian	217 (21)
Call City of Flagstaff Animal Control	844 (80)
Call Coconino County Public Health Services District	480 (45)
Call Game and Fish Department	159 (15)
Trap and relocate yourself	4 (0.3)
Take animal to animal control or humane society	43(4)
Take animal to your house	8 (0.7)
Kill the animal	29 (3)
Do not know	8 (0.7)
Response to seeing a sick wild animal†	
Nothing	45 (4)
Take the animal to a veterinarian	7 (0.6)
Call City of Flagstaff Animal Control	785 (74)
Call Coconino County Public Health Services District	608 (57)
Call Game and Fish Department	504 (48)
Trap and relocate yourself	3 (0.2)
Take animal to animal control or humane society	9 (0.8)
Take animal to your house	0
Kill the animal	65 (6)
Do not know	10 (0.9)
Response to being bitten or scratched by a domestic animal†	
Nothing	19 (2)
Wash wound with soap and water	740 (70)
Seek medical care at a hospital or clinic	757 (72)
Call City of Flagstaff Animal Control	544 (52)
Call Coconino County Public Health Services District	348 (33)
Call Game and Fish Department	93 (9)
Check animal’s vaccination history	563 (53)
Observe animal to see if it becomes rabid	178 (17)
Have animal tested for rabies	460 (44)
Kill the animal	22 (2)
Do not know	6 (0.6)
Response to being bitten or scratched by a wild animal†	
Nothing	2 (0.2)
Wash wound with soap and water	656 (62)
Seek medical care at a hospital or clinic	948 (90)
Call City of Flagstaff Animal Control	546 (52)
Call Coconino County Public Health Services District	521 (49)
Call Game and Fish Department	355 (34)
Check animal’s vaccination history	35 (3)
Observe animal to see if it becomes rabid	70 (7)
Have animal tested for rabies	362 (34)
Kill the animal	68 (6)
Do not know	8 (0.7)
Response to a wild nuisance animal on your property†	
Nothing	15 (1)
Call City of Flagstaff Animal Control	769 (73)
Call Coconino County Public Health Services District	439 (41)
Call Game and Fish Department	447 (42)
Call pest control company	105 (10)
Trap and relocate yourself	73 (7)
Kill the animal	49 (5)
Do not know	9 (0.8)
Relocated a wild nuisance animal on your property yourself	
No	932 (91)
Yes	95 (9)
If yes, distance away, miles,† n = 91	
<1	16 (18)
1–5	32 (35)
6–10	29 (32)
>10	23 (25)
Do not know	2 (2)
Animal relocated,† n = 94	
Bat	5 (5)
Skunk	53 (56)
Fox	4 (4)
Raccoon	17 (18)
Ringtail	0
Coyote	1 (1)
Mountain lion	0
Other	41 (44)
Wild animals on property in last 6 mo†	
Bat	254 (24)
Skunk	722 (68)
Fox	369 (35)
Sick wild animals on property in last 6 mo	
No	966 (94)
Yes	60 (6)
If yes, which animal,† n = 56	
Bat	1 (2)
Skunk	21 (38)
Fox	20 (36)
Other	21 (38)
Response to sick wild animal,† n = 53	
Nothing	19 (36)
Take the animal to a veterinarian	0
Call City of Flagstaff Animal Control	19 (36)
Call Coconino County Public Health Services District	9 (17)
Call Game and Fish Department	4 (8)
Trap and relocate yourself	1 (2)
Transport to animal control or humane society	1 (2)
Take animal to your house	0 (0)
Kill the animal	2 (4)
Do not know	0 (0)
Saw skunk in Flagstaff in last 6 mo	
No	215 (21)
Yes	790 (79)
Saw fox in Flagstaff in last 6 mo	
No	498 (50)
Yes	497 (50)
Saw oral rabies vaccination bait around property	
No	992 (96)
Yes	13 (1)
Do not know	24 (2)
Saw oral rabies vaccination bait in Flagstaff area	
No	950 (94)
Yes	32 (3)
Do not know	31 (3)
If saw a bait, touched or picked up bait, n = 47	
No	44 (94)
Yes	0 (0)
Do not know	3 (6)
Aware of current rabies outbreak in Flagstaff	
No	106 (10)
Yes	931 (90)
If yes, heard by,† n = 922	
TV	249 (27)
Radio	364 (39)
Newspaper/magazine	721 (78)
Family or friend	218 (24)
Notices/flyers on trailhead	341 (37)
Notices/flyers in county offices	67 (7)
Aware of rabies quarantine	
No	168 (16)
Yes	866 (84)
If yes, complied with quarantine	
No	120 (14)
Yes	712 (82)
The leash policy helps prevent exposures to rabid animals	
No	122 (12)
Yes	890 (88)

*The denominator used for each frequency was the total number of responses, from a total of 1,058 surveys, unless otherwise noted. †Survey question allowed for multiple choices.

**Table A2:** Univariate analysis of rabies knowledge in Flagstaff, in relation to pet ownership, dog ownership, and translocation with demographic and rabies-related characteristics*†

Characteristic	Knowledge of rabies‡				Pet owners				Dog owners				Translocators		
	Yes	No	OR (95% CI)		Yes	No	OR (95% CI)		Yes	No	OR (95% CI)		Yes	No	OR (95% CI)
Concern about rabies in Flagstaff															
Concerned	400	550	**3.20 (1.59–6.44)†**		622	313	1.20 (0.68–2.13)		478	472	1.09 (0.63–1.89)		90	833	1.76 (0.54–5.78)
Not concerned	10	44	Referent		33	20	Referent		26	28	Referent		3	49	Referent
Severity of rabies as a disease															
Severe	352	466	**2.55 (1.14–5.68)†**		543	263	1.22 (0.61–2.46)		412	406	1.35 (0.68–2.68)		73	723	1.62 (0.38–6.88)
Mild	8	27	Referent		22	13	Referent		15	20	Referent		2	32	Referent
Knowledge of rabies‡															
Yes	145	160	**1.52 (1.16–1.99)†**		214	91	**1.35 (1.01–1.80)†**		159	146	1.13 (0.87–1.48)		31	268	1.17 (0.74–1.83)
No	275	460	Referent		457	262	Referent		360	375	Referent		64	646	Referent
Knowledge of rabies in Flagstaff§															
Yes	NA	NA	NA		290	137	1.18 (0.91–1.54)		227	202	1.22 (0.96–1.57)		44	379	1.26 (0.82–1.92)
No	NA	NA	NA		394	220	Referent		301	328	Referent		51	553	Referent
Potential for contact with a sick domestic animal															
Yes	95	163	0.81 (0.61–1.09)		192	63	**1.82 (1.32–2.51)†**		153	105	**1.65 (1.24–2.20)†**		26	226	1.18 (0.73–1.89)
No	334	466	Referent		492	294	Referent		375	425	Referent		69	706	Referent
Potential for contact with a sick wild animal															
Yes	40	41	**1.47 (0.94–2.32)**		46	34	0.69 (0.43–1.09)		35	46	0.75 (0.47–1.18)		12	68	**1.84 (0.96–3.53)**
No	389	588	Referent		638	323	Referent		493	484	Referent		83	864	Referent
Potential for contact with a nuisance wild animal on property															
Yes	52	60	1.31 (0.88–1.94)		70	40	0.90 (0.60–1.36)		57	55	1.05 (0.71–1.55)		50	60	**16.15 (9.99–26.10)†**
No	377	569	Referent		614	317	Referent		471	475	Referent		45	872	Referent
Translocated a wild nuisance animal on their property															
Yes	44	51	1.26 (0.82–1.92)		53	41	**0.63 (0.41–0.97)†**		43	52	0.80 (0.52–1.22)		NA	NA	NA
No	379	553	Referent		616	301	Referent		475	457	Referent		NA	NA	NA
Aware of the quarantine in Flagstaff during 2009															
Yes	400	466	**4.69 (3.02–7.27)†**		596	268	**2.42 (1.73–3.38)†**		460	406	**1.89 (1.34–2.65)†**		81	761	1.21 (0.66–2.23)
No	26	142	Referent		80	87	Referent		63	105	Referent		13	148	Referent
Leash policy prevents pet exposure to rabid animals															
Yes	358	532	0.82 (0.56–1.20)		561	328	**0.34 (0.21–0.56)†**		415	475	**0.24 (0.15–0.37)†**		79	788	0.73 (0.40–1.34)
No	55	67	Referent		100	20	Referent		96	26	Referent		14	102	Referent
Pet owner															
Yes	290	394	1.18 (0.91–1.54)		NA	NA	NA		NA	NA	NA		53	616	**0.63 (0.41–0.97)†**
No	137	220	Referent		NA	NA	NA		NA	NA	NA		41	301	Referent
Dog owner															
Yes	227	301	1.22 (0.96–1.57)		NA	NA	NA		NA	NA	NA		43	475	0.80 (0.52–1.22)
No	202	328	Referent		NA	NA	NA		NA	NA	NA		52	457	Referent
Time lived in Flagstaff, y															
>10	333	432	**1.43 (1.07–1.90)†**		490	272	0.80 (0.59–1.07)		384	381	0.97 (0.74–1.28)		86	655	**3.73 (1.85–7.53)†**
<10	95	176	Referent		188	83	Referent		138	133	Referent		9	256	Referent
Sex															
F	230	370	**0.77 (0.59–0.99)†**		419	180	**1.51 (1.16–1.96)†**		315	285	1.19 (0.93–1.53)		45	538	**0.63 (0.41–0.97)†**
M	190	234	Referent		256	166	Referent		204	220	Referent		48	363	Referent
Age, y															
>60	154	225	0.96 (0.75–1.25)		196	183	**0.37 (0.28–0.48)†**		133	246	**0.36 (0.28–0.47)†**		42	326	1.42 (0.92–2.17)
<60	272	383	Referent		485	167	Referent		393	262	Referent		53	583	Referent
Education															
>College degree	336	423	**1.49 (1.11–2.00)†**		504	254	1.10 (0.82–1.48)		386	373	1.03 (0.78–1.37)		64	674	0.70 (0.44–1.10)
<College degree	89	167	Referent		164	91	Referent		128	128	Referent		30	220	Referent

*Results significant at p<0.1 are indicated in **boldface**. OR, odds ratio; NA, not applicable. †Results significant at p<0.05. ‡Knowledge of rabies is defined as ‘yes’ if the respondent 1) answered “yes” to bite, scratch, and saliva as modes of transmission and 2) “yes” to 1 or none of the following incorrect modes of transmission: touch, contact with blood, contact with urine or feces, contact with skunk spray. §Knowledge of rabies in Flagstaff is defined as “yes” if the respondent 1) knew about the outbreak in Flagstaff and 2) knew the 3 main animals that had rabies in Flagstaff (bat, skunk, fox).
